# Supplementary figures and images for: Human DC3 Antigen Presenting Dendritic Cells From Induced Pluripotent Stem Cells
Source: Front Cell Dev Biol. 2021 Jul 22;9:667304. doi: 10.3389/fcell.2021.667304 (PMC8339905; doi:10.3389/fcell.2021.667304)

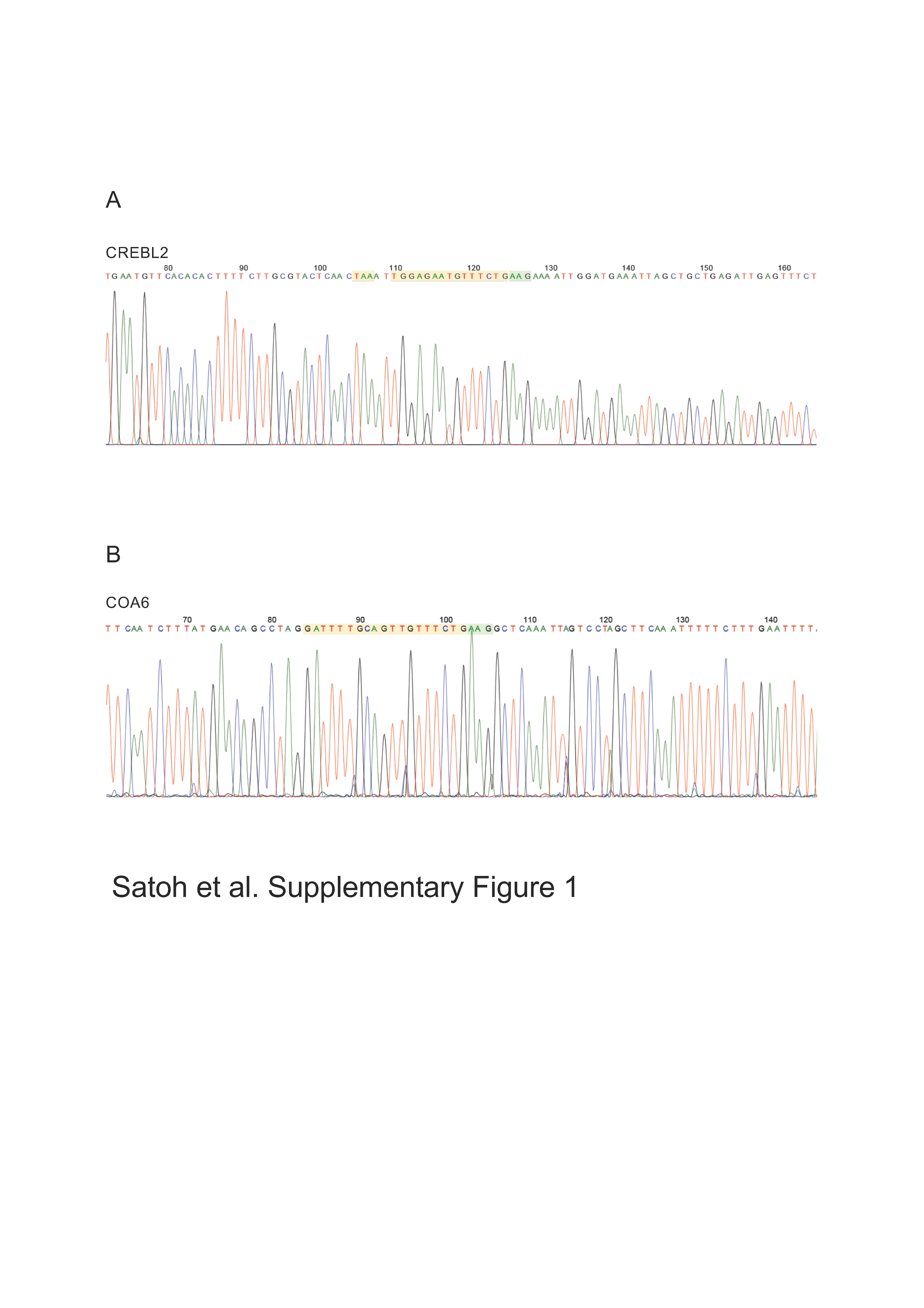

Supplement: Supplementary Figure 1 — DNA sequence of potential CRISPR/Cas9 off-target genes CREBL2 and COA6. The potential CRISPR/Cas9 off-target genes cAMP responsive element binding protein like 2 (CREBL2) and cytochrome C oxidase assembly factor 6 (COA6) were analyzed by DNA sequencing (A,B, respectively) and no off-target effects were found. CRISPR/Cas9 gRNA sequence targeting JAK2 (light orange box); PAM sequence (light green box). [file Image_1.TIFF]

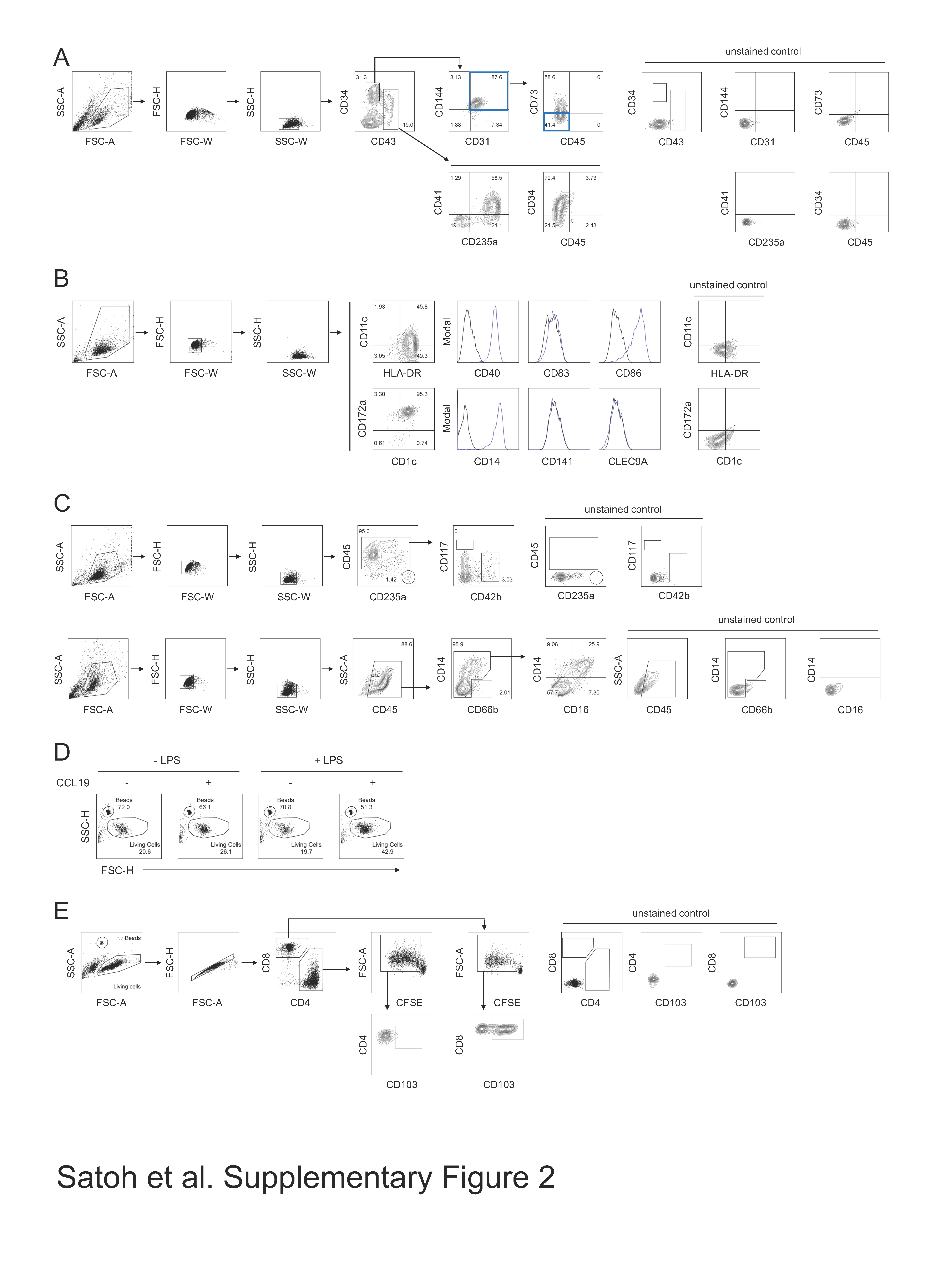

Supplement: Supplementary Figure 2 — Gating strategies for flow cytometry analysis. (A) Gating strategy for CD34+ CD31+ CD144+ CD43– CD45– CD73– HE (blue boxes) and CD43+ CD34low/− HPC in Figures 1B,2A. (B) Gating strategy for HLA-DR+ DC and the DC subsets cDC1 (CD141 and CLEC9A) and cDC2 (CD1c and CD172a) and for the co-stimulatory molecules CD40, CD83 and CD86 in Figures 1C,F,G, 2B,C and Supplementary Figures 4B–D. (C) Gating strategy for CD235a/glycophorin A+ erythrocytes, CD42b+ megakaryocytes and CD14+, CD16+, and CD66b+ myeloid cells (monocytes, makrophages and granulocytes, respectively) in Figures 3A,B. (D) Gating strategy for DC migration toward CCL19 chemokine in Figure 4A and Supplementary Figure 5A. (E) Gating strategy for CD4+ and CD8+ T cells and for CD103 in T cell activation assays in Figure 4B. [file Image_2.TIFF]

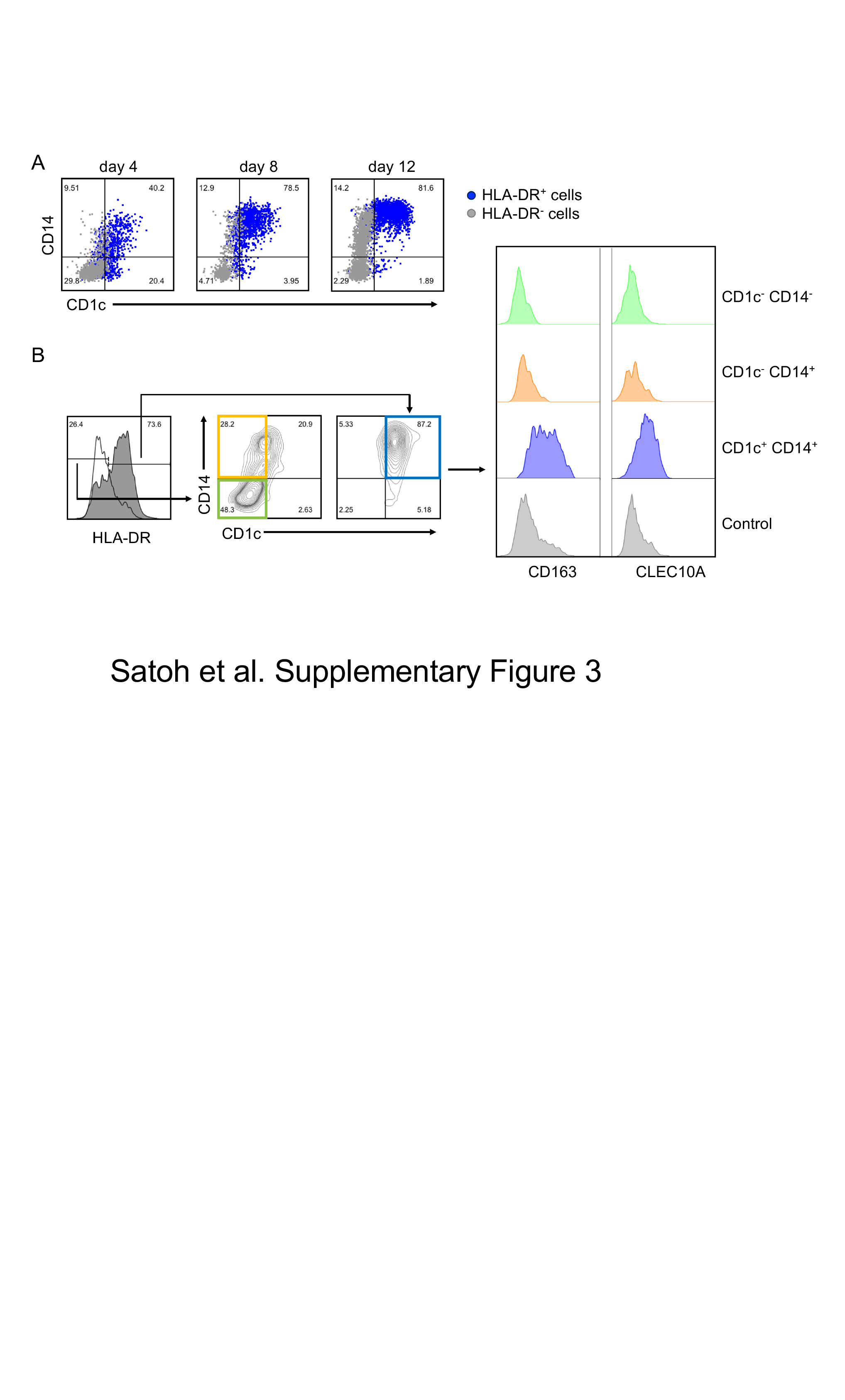

Supplement: Supplementary Figure 3 — (A) Kinetics of emerging HLA-DR+ CD1c+ CD14+ cells of healthy donor during DC differentiation at days 4, 8 and 12 by flow cytometry. (B) Representative flow cytometry analysis of CD163 and CLEC10A on HLA-DR+ CD1c+ CD14+ cells of healthy donor on days 7–8 of DC differentiation. Control, unstained cells. n = 2. [file Image_3.TIF]

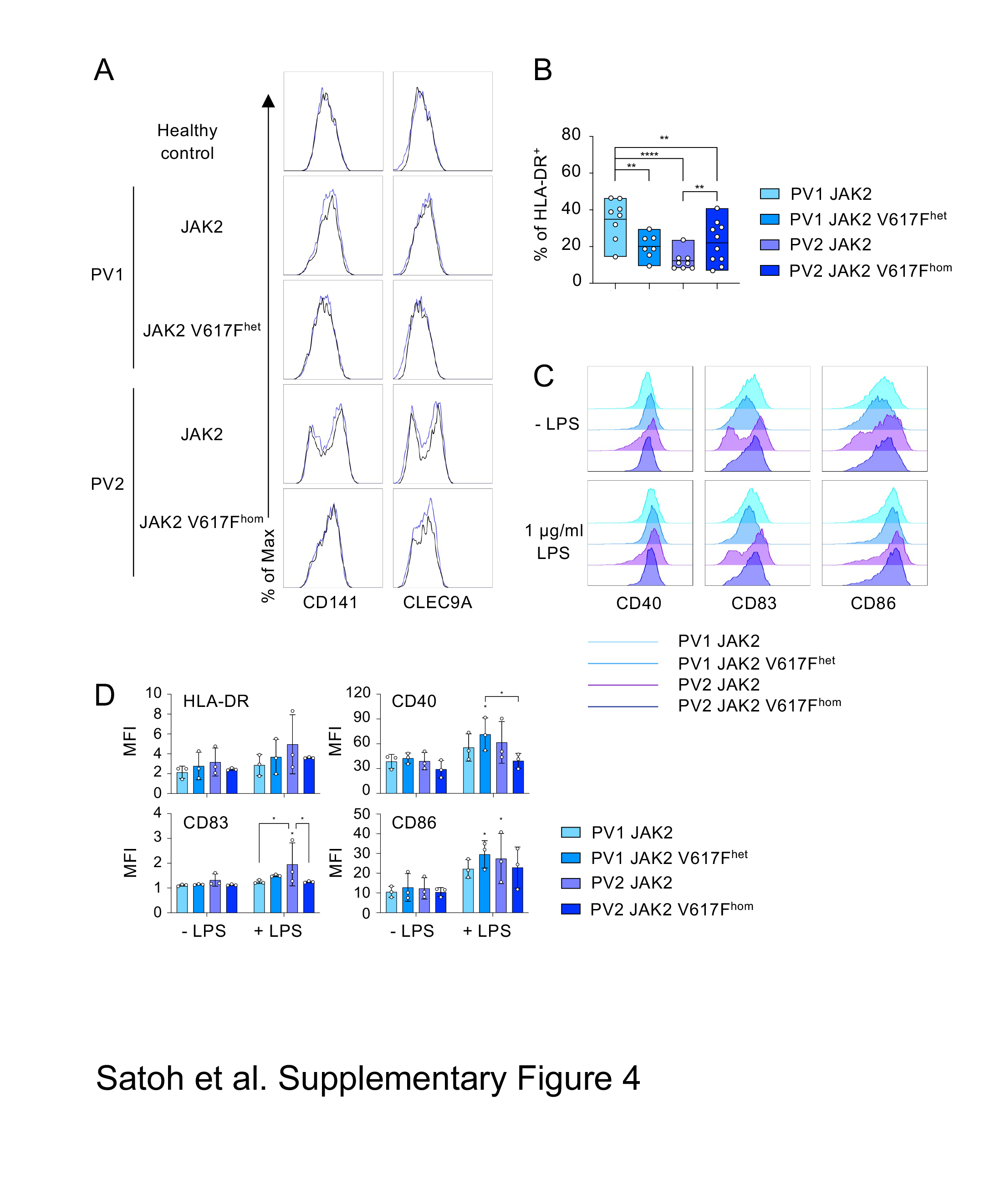

Supplement: Supplementary Figure 4 — (A) Representative flow cytometry analysis of CD141 and CLEC9A on HLA-DR+ cells on day 8–9 of DC differentiation. JAK2 genotypes of patients PV1 and PV2, and healthy donor (healthy control) are as in Figure 3A. Blue lines and black lines in histograms represent stained and unstained cells, respectively. n = 2–3. (B) The percentage of HLA-DR+ cells on day 8–9 of DC differentiation for JAK2 V617Fhet and JAK2 V617Fhom cells of patients PV1 and PV2, and for cells without mutation (JAK2). n = 7–10, line represents mean; ∗p < 0.05, ∗∗p < 0.005, ****p < 0.0001, one-way ANOVA with uncorrected fisher’s LSD test. (C) Representative flow cytometry analysis of CD40, CD83, and CD86 on unstimulated and LPS-stimulated CD1c+ HLA-DR+ cells on day 8–9 + 1 of DC differentiation. JAK2 genotypes of patients PV1 and PV2 are as in (B). n = 3. (D) Expression of DC activation markers CD40, CD83 and CD86 on unstimulated and LPS-stimulated CD1c+ HLA-DR+ cells of (C). MFI values were normalized to unstained cells. JAK2 genotypes of patients PV1 and PV2 are as in (B). n = 3, mean ± SD; ∗p < 0.05, two-way ANOVA with uncorrected fisher’s LSD test between unstimulated and LPS-stimulated cells for each JAK2 V617F genotype or as indicated. [file Image_4.TIF]

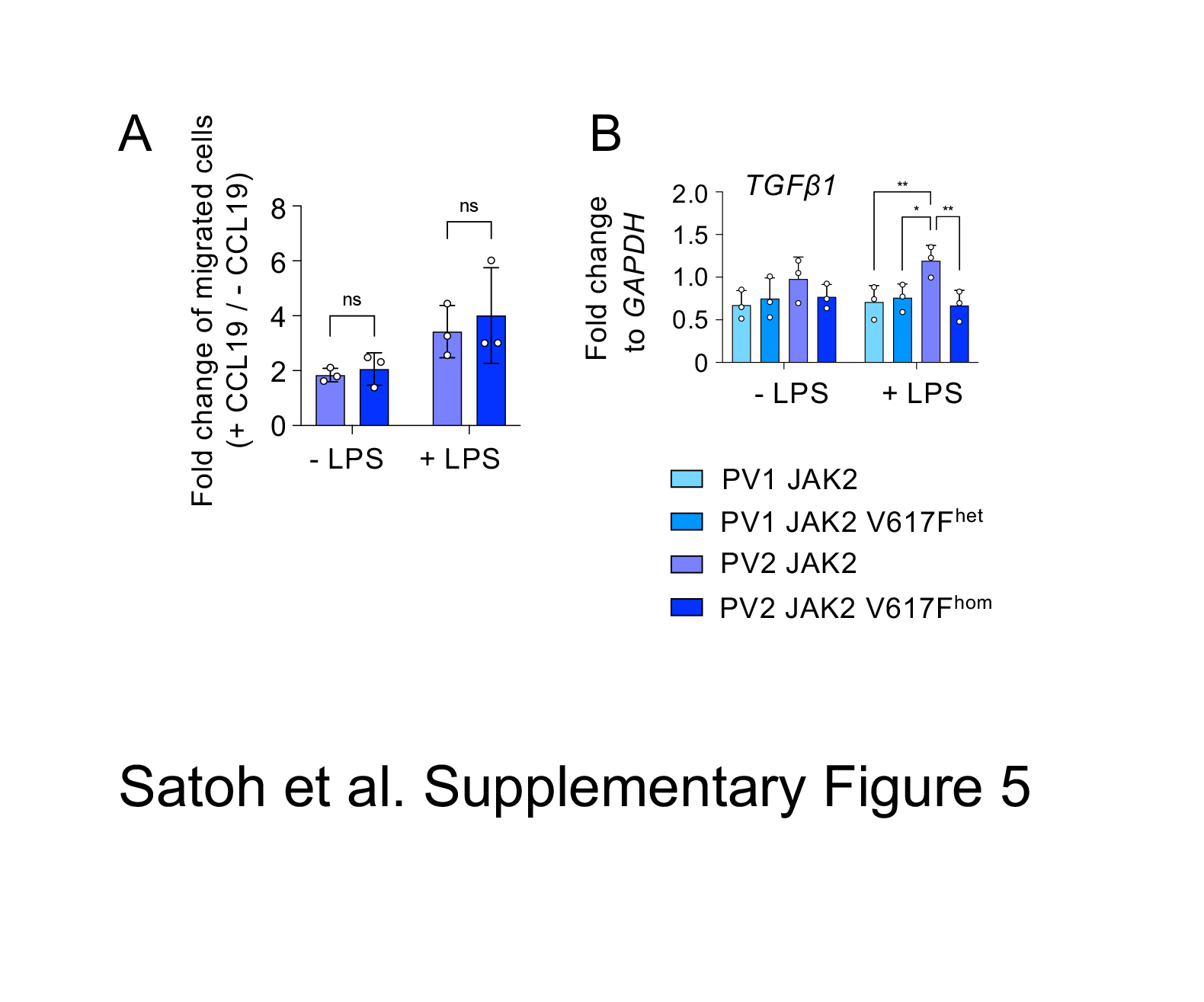

Supplement: Supplementary Figure 5 — (A) Chemotaxis assay toward CCL19 of JAK2 V617Fhom and JAK2 control CD1c+ HLA-DR+ cells as in Figure 4A. Numbers of migrated cells were determined by flow cytometry and the fold change to the cell number without CCL19 is shown. n = 3, mean ± SD; ns, not significant. (B) TGFβ1 expression in unstimulated and LPS-stimulated HLA-DR+ cells of Supplementary Figure 4C determined by RT-qPCR analysis. Values were normalized to GAPDH and 2–ΔCt values were shown. n = 3, mean ± SD; ∗p < 0.05, ∗∗p < 0.005, one-way ANOVA with uncorrected fisher’s LSD test between unstimulated and LPS-stimulated cells for the various JAK2 V617F genotypes as indicated. [file Image_5.TIF]
