# Supplementary material for: Human DC3 Antigen Presenting Dendritic Cells From Induced Pluripotent Stem Cells
Source: Front Cell Dev Biol. 2021 Jul 22;9:667304. doi: 10.3389/fcell.2021.667304 (PMC8339905; doi:10.3389/fcell.2021.667304)
Supplement: Supplementary file 6 [file Table_1.DOCX]

|  | Sequence | |
| --- | --- | --- |
| crRNA | 5’-AATTATGGAGTATGTTTCTG-3’ | |
| donor template | 5’-AGCAAGTATGATGAGCAAGCTTTCTCACAAGCATTTGGTTTTAAATTATGGAGTGTGTG  TCTGTGGAGACGAGAGTAAGTAAAACTACAGGCTTTCTAATGCCTTTCTCAGAGCATCTGT-3’ | |
|  | Sequence (Forward) | Sequence (Reverse) |
| JAK2 | 5’-TCCTCAGAACGTTGATGGCAG-3’ | 5’-ATTGCTTTCCTTTTTCACAAGAT-3’ |
| JAK2 WT | 5’-GCATTTGGTTTTAAATTATGGAGTATATG-3’ | 5’-GTTTTACTTACTCTCGTCTCCACAAAC-3’ |
| JAK2 V617F |  | 5’-GTTTTACTTACTCTCGTCTCCACAAAA-3’ |
| CREBL2 | 5’-CTTTCAGGCATTTCAGCAGC-3’ | 5’-CATATCCAGGGCTGAACCAG-3’ |
| COA6 | 5’-TAACCTGCACGTTGTGCAC-3’ | 5’-GGGAACAAGTATTGATGTTCAC-3’ |

Supplementary Table 1: Sequences of crRNA and donor template for CRISPR/Cas9 editing, JAK2 sequencing primers (JAK2) and JAK2 V617F allele specific PCR primers (JAK2 WT and JAK2 V617F) and sequencing primer for CREBL2 and COA6 off-target analysis.

JAK2 primer sequences are from Jones, A.V., Kreil, S., Zoi, K., Waghorn, K., Curtis, C., Zhang, L., et al. (2005). Widespread occurrence of the JAK2 V617F mutation in chronic myeloproliferative disorders. *Blood* 106**,** 2162-2168. doi: 10.1182/blood-2005-03-1320.
