# Supplementary material for: Human DC3 Antigen Presenting Dendritic Cells From Induced Pluripotent Stem Cells
Source: Front Cell Dev Biol. 2021 Jul 22;9:667304. doi: 10.3389/fcell.2021.667304 (PMC8339905; doi:10.3389/fcell.2021.667304)
Supplement: Supplementary file 7 [file Table_2.docx]

| Antibody | Source | Identifier | Dilution |
| --- | --- | --- | --- |
| Mouse anti-human CD1c-Biotin (clone AD5-8E7) | Miltenyi Biotec | Cat# 130-113-300 | (1:100) |
| Mouse anti-human CD4-PE-Cy7 (clone SK3) | BD | Cat# 560909 | (1:100) |
| Mouse anti-human CD8-APC (clone SK1) | BD | Cat# 345775 | (1:100) |
| Mouse anti-human CD11c-PE-Cy7 (clone 3.9) | eBioscience | Cat# 25-0116-42 | (1:200) |
| Mouse anti-human CD14-PE (clone MøP9) | BD | Cat# 562691 | (1:100) |
| Mouse anti-human CD14-APC-Cy7 (clone MøP9) | BD | Cat# 561709 | (1:200) |
| Mouse anti-human CD16-APC (clone B73.1) | BD | Cat# 561304 | (1:100) |
| Mouse anti-human CD31-Biotin (clone AC128) | Miltenyi Biotec | Cat# 130-098-679 | (1:100) |
| Mouse anti-human CD34-APC (clone 581) | BioLegend | Cat# 343513 | (1:200) |
| Mouse anti-human CD40-APC/Fire750 (clone 5C3) | BioLegend | Cat# 334344 | (1:200) |
| Mouse anti-human CD41-PE-Cy7 (clone HIP8) | BioLegend | Cat# 303718 | (1:1000) |
| Mouse anti-human CD42b-APC (clone HIP1) | BioLegend | Cat# 303912 | (1:100) |
| Mouse anti-human CD43-FITC (clone 1G10) | BD | Cat# 555475 | (1:100) |
| Mouse anti-human CD45-APC-Vio770 (clone 5B1) | Miltenyi Biotec | Cat# 130-113-115 | (1:200) |
| Mouse anti-human CD45-V450 (clone 2D1) | BD | Cat# 642275 | (1:100) |
| Mouse anti-human CD66b-PE (clone G10F5) | BD | Cat# 561650 | (1:100) |
| Mouse anti-human CD66b-PerCP-Cy5.5 (clone G10F5) | BD | Cat# 562254 | (1:100) |
| Mouse anti-human CD73-PE (clone AD2) | Immunotools | Cat# 21270734X2 | (1:100) |
| Mouse anti-human CD83-PE (clone HB15e) | eBioscience | Cat# 12-0839-71 | (1:100) |
| Mouse anti-human CD86-APC (clone IT2.2) | BioLegend | Cat# 305411 | (1:200) |
| Mouse anti-human CD103-BV421 (clone Ber-ACT8) | BioLegend | Cat# 350213 | (1:100) |
| Mouse anti-human CD117-PE-Cy7 (clone 104D2) | eBioscience | Cat# 25-1178-42 | (1:100) |
| Mouse anti-human CD141-VioBlue (clone AD-14H12) | Miltenyi Biotec | Cat# 130-113-882 | (1:200) |
| Human anti-human CD144-PE-Vio770 (clone REA199) | Miltenyi Biotec | Cat# 130-100-720 | (1:100) |
| Mouse anti-human CD163-BV421 (clone GHI/61) | BD | Cat# 566277 | (1:200) |
| Mouse anti-human CD172a/b-APC/Fire750 (clone SE5A5) | BioLegend | Cat# 323817 | (1:200) |
| Mouse anti-human CD235a-PE (clone HIR2) | eBioscience | Cat# 12-9987-82 | (1:1000) |
| Human anti-human CLEC9A-APC (clone REA976) | Miltenyi Biotec | Cat# 130-116-418 | (1:200) |
| Mouse anti-human CLEC10A-APC (clone H037G3) | BioLegend | Cat# 354705 | (1:200) |
| Mouse anti-human HLA-DR-FITC (clone LN3) | eBioscience | Cat# 11-9956-42 | (1:200) |
| Streptavidin-PE-Cy5.5 | eBioscience | Cat# 35-4317-82 | (1:500) |

Supplementary Table 2: Antibodies used for FACS in this study
