# Supplementary material for: Human DC3 Antigen Presenting Dendritic Cells From Induced Pluripotent Stem Cells
Source: Front Cell Dev Biol. 2021 Jul 22;9:667304. doi: 10.3389/fcell.2021.667304 (PMC8339905; doi:10.3389/fcell.2021.667304)
Supplement: Supplementary file 8 [file Table_3.DOCX]

| Gene | Sequence (Forward) | Sequence (Reverse) | References |
| --- | --- | --- | --- |
| CCR7 | 5'-GTGGTGGCTCTCCTTGTCAT-3' | 5'-CCAGTAGGCCCACGAAACAA-3' |  |
| CD14 | 5'-CCGCTGTGTAGGAAAGAAGCTA-3' | 5'-TCTTCATCGTCCAGCTCACAAG-3' |  |
| CD31 | 5'-GAGTCCTGCTGACCCTTCTG-3' | 5'-ATTTTGCACCGTCCAGTCC-3' | [1] |
| CD34 | 5'-TGGACCGCGCTTTGCT-3' | 5'-CCCTGGGTAGGTAACTCTGGG-3' | [1] |
| CD40 | 5'-GAACCTCTCACTTCACCCTGGA-3' | 5'-GGATCGGAAGGTCTGGTGGATA-3' |  |
| CD45 | 5'-TCAGCCTTGCACACCACAGCTC-3' | 5'-AAATGACAGCGCTTCCAGAAGGGC-3' |  |
| CD80 | 5'-GAAGGGAAAGTGTACGCCCT-3' | 5'-TACGTAAAGGGCAAGGTGGG-3' |  |
| CD83 | 5'-GGAGAAGAAGTCAGAGAGGGTG-3' | 5'-TGGAGAAAGCAGACACCAGAAG-3' |  |
| CD86 | 5'-AAGCAGCCAAAATGGATCCCC-3' | 5'-GGCAGGTCTGCAGTCTCATTG-3' |  |
| CD144 | 5'-TGGAGAAGTGGCATCAGTCAACAG-3' | 5'-TCTACAATCCCTTGCAGTGTGAG-3' | [2] |
| CD163 | 5’-TCTTGGGACTTGGACGATGC-3’ | 5’-GCTTTTTGTGGGGTTTTCTGC-3’ |  |
| CSF1R | 5'-CCTGAAGGTGGCTGTGAAGATG-3' | 5'-GCTCCCAGAAGGTTGACGATG-3' | [3] |
| CSF2RA | 5'-CTACTCAGAAGCGGGAGTCT-3' | 5'-CGTACAGGGTTCTACGCAAA-3' |  |
| EOMES | 5'-ATCATTACGAAACAGGGCAGGC-3' | 5'-CGGGGTTGGTATTTGTGTAAGG-3' | [4] |
| GAPDH | 5'-GAAGGTGAAGGTCGGAGTC-3' | 5'-GAAGATGGTGATGGGATTTC-3' | [5] |
| GATA1 | 5'-GGGATCACACTGAGCTTGC-3' | 5'-ACCCCTGATTCTGGTGTGG-3' | [6] |
| GATA2 | 5'-GGGCTAGGGAACAGATCGACG-3' | 5'-GGGCTAGGGAACAGATCGACG-3' | [6] |
| IL-6 | 5'-TCCAAAGATGTAGCCGCCC-3' | 5'-CAGTGCCTCTTTGCTGCTTTC-3' |  |
| IL-8 | 5'-GGGCCAAGAGAATATCCGAACT-3' | 5'-CTGTGAGGTAAGATGGTGGCTA-3' |  |
| IL-10 | 5'-CCAAGACCCAGACATCAAGGC-3' | 5'-CATTCTTCACCTGCTCCACGG-3' |  |
| IRF4 | 5'-CCATGACAACGCCTTACCCT-3' | 5'-ATTGGTACGGGATTTCCGGG-3' |  |
| IRF8 | 5'-AGCATGTTCCGGATCCCTTG-3' | 5'-AGTGGCTGGTTCAGCTTTGT-3' |  |
| ITGAX | 5'-CCAGATCACCTTCTTGGCTACC-3' | 5'-TCCCTCTGTCCCAGGTTATTGA-3' |  |
| KDR | 5'-CCTCTACTCCAGTAAACCTGATTGGG-3' | 5'-TGTTCCCAGCATTTCACACTATGG-3' | [2] |
| MHC2 | 5'-ATGCACCATTGGCTCCTGTTT-3' | 5'-TAGCACTGGAGTGGCAGATAG-3' |  |
| MIXL1 | 5'-GTACCCCGACATCCACTTGC-3' | 5'-AGGATTTCCCACTCTGACGC-3' |  |
| NANOG | 5'-CAGAAGGCCTCAGCACCTAC-3' | 5'-ATTGTTCCAGGTCTGGTTGC-3' | [7] |
| OCT3/4 | 5'-GGGGGTTCTATTTGGGAAGGTA-3' | 5'-ACCCACTTCTGCAGCAAGGG-3' | [8] |
| PD-L1 | 5'-AAGGCCGAAGTCATCTGGACAA-3' | 5'-TCTCCTCTCTCTTGGAATTGGTGG-3' |  |
| PU.1 | 5'-ACGGATCTATACCAACGCCA-3' | 5'-GGGGTGGAAGTCCCAGTAAT-3' | [6] |
| SCL | 5'-GGATGCCTTCCCTATGTTCA-3' | 5'-AGGCGGAGGATCTCATTCTT-3' | [9] |
| SIRPA | 5'-ATATTGTGGTGGGTGTGGTGTG-3' | 5'-TTTCTCTGGCATTCTTCTCGGG-3' |  |
| SOCS1 | 5'-GCATTAACTGGGATGCCGTGT-3' | 5'-ATGAAGAGGTAGGAGGTGCGAG-3' |  |
| SOCS2 | 5'-CGCAGACAAGGAGATGAGTTTC-3' | 5'-CCTCTACCCTACCTGGGATTCT-3' |  |
| SOCS3 | 5'-GCTCCTTTGTGGACTTCACGG-3' | 5'-CGGGAAACTTGCTGTGGGTG-3' |  |
| T | 5'-CAGTGGCAGTCTCAGGTTAAGAAGGA-3' | 5'-CGCTACTGCAGGTGTGAGCAA-3' | [10] |
| TGFB1 | 5'-GAGCCTGAGGCCGACTACTA-3' | 5'-CTTCTCGGAGCTCTGATGTGTT-3' |  |
| TNFa | 5'-ACTTTGGAGTGATCGGCCC-3' | 5'-CATTGGCCAGGAGGGCATT-3' |  |

Supplementary Table 3: Primers used for RT-qPCR in this study.

**Reference list for primers**

1. Zambidis, E. T., Peault, B., Park, T. S., Bunz, F., and Civin, C. I. (2005). Hematopoietic differentiation of human embryonic stem cells progresses through sequential hematoendothelial, primitive, and definitive stages resembling human yolk sac development. *Blood* 106, 860–870. doi:10.1182/blood-2004-11-4522.
2. Kennedy, M., D’Souza, S. L., Lynch-Kattman, M., Schwantz, S., and Keller, G. (2007). Development of the hemangioblast defines the onset of hematopoiesis in human ES cell differentiation cultures. *Blood* 109, 2679–2687. doi:10.1182/blood-2006-09-047704.
3. Wang, X. S., Gong, J. N., Yu, J., Wang, F., Zhang, X. H., Yin, X. L., et al. (2012). MicroRNA-29a and microRNA-142-3p are regulators of myeloid differentiation and acute myeloid leukemia. *Blood* 119, 4992–5004. doi:10.1182/blood-2011-10-385716.
4. Teo, A. K. K., Arnold, S. J., Trotter, M. W. B., Brown, S., Ang, L. T., Chng, Z., et al. (2011). Pluripotency factors regulate definitive endoderm specification through eomesodermin. *Genes Dev* 25, 238–250. doi:10.1101/gad.607311.
5. Qin, J., Li, W. Q., Zhang, L., Chen, F., Liang, W. H., Mao, F. F., et al. (2010). A stem cell-based tool for small molecule screening in adipogenesis. *PLoS ONE* 5. doi:10.1371/journal.pone.0013014.
6. Szabo, E., Rampalli, S., Risueño, R. M., Schnerch, A., Mitchell, R., Fiebig-Comyn, A., et al. (2010). Direct conversion of human fibroblasts to multilineage blood progenitors. *Nature* 468, 521–526. doi:10.1038/nature09591.
7. Zhang, J., Wilson, G. F., Soerens, A. G., Koonce, C. H., Yu, J., Palecek, S. P., et al. (2009). Functional cardiomyocytes derived from human induced pluripotent stem cells. *Circ Res* 104, 30–41. doi:10.1161/CIRCRESAHA.108.192237.
8. Tran, T. H., Wang, X., Browne, C., Zhang, Y., Schinke, M., Izumo, S., et al. (2009). Wnt3a-induced mesoderm formation and cardiomyogenesis in human embryonic stem cells. *Stem Cells* 27, 1869–1878. doi:10.1002/stem.95.
9. Dravid, G., Zhu, Y., Scholes, J., Evseenko, D., and Crooks, G. M. (2011). Dysregulated gene expression during hematopoietic differentiation from human embryonic stem cells. *Mol Ther* 19, 768–781. doi:10.1038/mt.2010.281.
10. Yang, L., Soonpaa, M. H., Adler, E. D., Roepke, T. K., Kattman, S. J., Kennedy, M., et al. (2008). Human cardiovascular progenitor cells develop from a KDR+ embryonic-stem-cell-derived population. *Nature* 453, 524–528. doi:10.1038/nature06894.
